# The Microfloral Analysis of Secondary Caries Biofilm around Class I and Class II Composite and Amalgam Fillings

**DOI:** 10.1186/1471-2334-10-241

**Published:** 2010-08-17

**Authors:** Si-su Mo, Wei Bao, Guang-yun Lai, Jun Wang, Ming-yu Li

**Affiliations:** 1 Shanghai Key Laboratory of Stomatology, Shanghai Research Institute of Stomatology, Ninth People's Hospital, Faculty of Medicine, Shanghai Jiao Tong University, 639 Zhizaoju Road, 200011 Shanghai, China; 2 Department of Stomatology, Shanghai Huashan Hospital, 12 Wulumuqi Zhong Road, 200042 Shanghai, China

## Abstract

**Background:**

Secondary caries is responsible for 60 percent of all replacement restorations in the typical dental practice. The diversity of the bacterial sources and the different types of filling materials could play a role in secondary caries. The aim of this study was to determine and compare the microbial spectrum of secondary caries biofilms around amalgam and composite resin restorations.

**Methods:**

Clinical samples were collected from freshly extracted teeth diagnosed with clinical secondary caries. Samples were categorized into four groups according to the types of restoration materials and the classification of the cavity. Biofilms were harvested from the tooth-restoration interface using a dental explorer and after dilution were incubated on special agars. The bacteria were identified using the biochemical appraisal system. Statistical calculations were carried out using SPSS11.5 software to analyze the prevalence of the bacteria involved in secondary caries.

**Results:**

Samples from a total of four groups were collected: two groups were collected from amalgam restorations, each had 21 samples from both Class I and Class II caries; and the other two groups were from composite resin restorations, each had 13 samples from both class I and class II caries. Our results showed: (1) Anaerobic species were dominant in both restoration materials. (2) In terms of the types of individual bacteria, no significant differences were found among the four groups according to the geometric mean of the detected bacteria (P > 0.05). However, there were significant differences among the detected bacteria within each group (P < 0.05). The composition of each bacterium had no statistical difference among the four groups (P > 0.05), but showed significant differences among the detected bacteria in each group (P < 0.05). (3) Among the four groups, there were no significant differences for the detection rate of each bacterium (P > 0.05), however, the detection rate of each bacterium within each group was statistically different among the detected bacteria (P < 0.05).

**Conclusions:**

The proportion of obligatory anaerobic species was much greater than the facultative anaerobic species in the biofilm of secondary caries. Statistically, the materials of restoration and the location of secondary caries did not show any significant effects on the composition of the microflora.

## Background

The term "secondary caries" or "recurrent caries" denotes caries at the margin of the tooth restorations, which is the most important reason for the failure of fillings [1-4]. Secondary caries is responsible for 60 percent of all restoration replacement in the typical dental practice. The bacteria present in the dental plaque that are involved in the etiology of primary caries most likely also play a major role in the development of secondary caries [5]. It has been reported that the material properties of the dental restorations influence plaque accumulation and development of secondary caries [6,7]. However, in the culture studies of Kidd et al., they found no significant differences in the microflora composition in plaque samples taken from sites with primary or recurrent caries. Similarly, some studies also failed to find any significant association between the microbial flora among different dental materials, several studies reported specific microbial spectrum profiles or found a correlation between the roughness of dental materials and the accumulation of bacteria [8-10]. This indicated that the antibacterial effects of metal ions from dental materials could play a role in secondary caries. Svanberg et al. detected much higher total colony-forming unit (cfu) counts for mutans streptococci at margins of composite fillings than that of comparable amalgam fillings [11]. The amount of plaques and the degree of cariogenicity at restoration margins depend on the restorative material [11,12]. These findings indicated that resin based materials accumulate more plaques, which are more cariogenic than amalgam, silicate cement, and glass ionomer materials. On the other hand, polymerization shrinkage and the load of chewing pressure often result in cracking and microleakage of the composites [13]. This marginal gap could be an ecological niche for microorganisms [14], especially because composites do not have the antibacterial effects of, for example, Hg-ions in amalgam [15]. Moreover, Matasa described a "microbial attack" on composites used as bonding adhesives for orthodontic applications [16]. It was believed that microleakage was also associated with the development of recurrent caries [17,18] but this theory has been challenged. The bulk of available evidence indicates that there is no relationship between the development of recurrent caries and the size of the crevice at the tooth restoration interface [19-24], apart from cases of macroleakage in which the crevice exceeded 250 μm or 400 μm [25]. Thus, recurrent caries does not develop as a result of microleakage along the tooth-restoration interface, but at a surface lesion similar to primary carious lesions on smooth surfaces [24,4]. The presences of overhangs, even the clinically hard-to-detect minor overhangs, predispose a patient to the development of recurrent caries, which indicated that plaque accumulation is an important predisposing factor in the development of recurrent caries [24].

Most studies have concentrated on aerobic bacteria around the restorations, but little is known about the anaerobic bacteria. Although these gram-negative anaerobic bacteria have been shown to associate with periodontal diseases, Christian Splieth et al. found that anaerobic gram-negative bacteria associated with periodontal diseases were predominant in secondary caries in composite fillings [26]. These bacteria were similar to the bacteria spectrum of root canal infection such as *Fusobacterium, Veillonella, Prevotella*, etc. Therefore, we infer anaerobic bacteria may also play an important role in formation of secondary caries and we have focused on some of these anaerobic periodontal bacteria in this study.

The aim of this study was to determine and compare the microbial spectrum around composite and amalgam fillings with particular attention to the anaerobic flora.

## Methods

### Sample collection

This study was approved by the Ethics Committee of Ninth People's Hospital, Medical College, Shanghai Jiao Tong University, China. Informed consent has been acquired from all patients participating in this study. The patients, who contributed teeth to this study, had mostly had periodontal diseases. Biofilm samples were collected from freshly extracted teeth that had been diagnosed with secondary caries upon visual inspection by experienced dentists according to the modified criteria of the California Dental Association [27-29]. Samples were collected and categorized into four groups according to the restoration materials and G. V. Black's cavity classification: Group 1 were samples collected from amalgam-filled class I cavities, Group 2: class II cavities with amalgam, Group 3: class I cavities with composite resin, and Group 4: class II cavities with composite resin. The teeth were routinely extracted and rinsed thoroughly with deionised water. The biofilm samples were collected with a dental explorer and immediately transferred into vials containing 1 ml transport fluid of prereduced thioglycollate medium (Difco, Detroit, Mich., USA).

### Bacterial culture procedures

Samples were vortexed for 10 seconds. After 10^5 ^times dilution, samples were plated on total 9 special agar plates (as shown in Table 1) in triplicate and cultured at 37°C. For each plate 50 μl of diluted sample were used. Plates for anaerobic bacteria CDC, KVLB, V-Rogosa, FS, and PS were incubated in an anaerobic work station (BUG BOX DUAL, Fuskinn, England) in 80% N_2_, 10% H_2 _and 10% CO_2 _at 37°C for at least 4 days. Plates for aerobic bacteria TSA, MS, CFAT, and L-Rogosa were incubated in an anaerobic work station in 90% N_2 _and 10% CO_2 _at 37°C for at least 2 days. After incubation, colonies formed on the plates were initially identified with the morphology using a stereomicroscope, and the cfu counts were recorded accordingly. The further identification of the bacteria was conducted as the following: the bacteria were firstly checked with Gram's staining, their aerotolerance and antibiotics sensitivity. Then biochemical analysis including the fermentation of carbohydrates and production of indole and nitrate was performed using a kit from KLOBME (Key Laboratory of Oral Biomedical Engineering, Ministry of Education, Chengdu, China). Reference species in the biochemical analysis were listed in the following: *Streptococci mutans *NCTC Ingbritt, *Actinomyces viscosus *ATCC 19246, *Lactobacilli acidophilus *ATCC 4356, *Neisseriae mucosa *ATCC 49233, *Prevotella corporis *ATCC 3354, *Prevotella melaninogenica *ATCC 33563, *Porphyromonas gingivalis *ATCC 33277, *Veillonella parvula *ATCC 10790, *Fusobacterium nucleatum *ATCC 23276, *Peptostreptococcus anaerobius *ATCC 27337, and *Capnocytophaga ochracea *ATCC 33596.

**Table 1:** Media used in this study

Media	Application
Tryptic Soy Agar plate (TSA)	universal for aerobic bacteria
Mitis-Salivarius Bacitracin agar (MSB)	Oral streptococci-selective
Cadmium fluoride acriflavin tellurite (CFAT)	Actinomyces-selective
L-Rogosa selective blood agar plate	Lactobacilli-selective
Anaerobic CDC blood agar plate	universal for obligative anaerobic bacteria
kanamycin-vancomycin laked blood plate (KVLB)	Porphyromonas-selective
V-Rogosa blood agar plate	Veillonella-selective
FS blood agar plate	Fusobacterium-selective
PS blood agar plate	Peptostreptococcus-selective

### Statistical analysis

Bacteria colony-forming units (cfu) were presented as geometric averages. The composition of each bacterium in the sample plaque was calculated as the percentage ratio of its cfu count formed on the selective culture plate compared with the total cfu formed in the universal plates. The detection rate of each bacterium in each group was calculated as the percentage ratio of the number of sample which we can idenfied one kind of the bacteria from devided by the number of sample in each group.

The prevalence of the bacteria involved in secondary caries was analyzed using SPSS11.5 software. Total cfu of each sample was presented as a log phase to accommodate the normal distributions. The differences of Geometric averages of cfu and composition of each bacterium in the four groups were compared by the two-way ANOVA analysis using the Bonferroni method. For the differences of detection rate of the bacteria among the four groups, Chi-Square Test was used for analysis.

## Results

The microflora of secondary caries biofilm predominantly included *Prevotella, Veillonella, Lactobacilli, Streptococci mutans, Neisseriae*, and *Actinomyces; *followed by *Peptostreptococcus, Fusobacterium*, and *Porphyromonas gingivalis; *and occasionally *Capnocytophaga *was detected. The ratios of aerobic to anaerobic flora were comparable between composite resin and amalgam groups, 37.64%/62.36% and 38.09%/61.91%, respectively. These ratios were also comparable between class I and class II caries, 37.12%/62.87% and 38.60%/61.40%, respectively (data not shown). The composition of each bacterium had no statistical difference among the four groups (P > 0.05), however, significant differences were found among the detected bacteria in each group (P < 0.05) as shown in Table 2. There were no significant differences among the four groups for the detection rate of each bacterium (P > 0.05), but the detection rate among the detected bacteria had statistical differences within each group (P < 0.05) as shown in Table 3.

**Table 2:** The log colony-forming units per ml from four groups (logXG ± SD)

Bacteria	class I cavities of amalgam (n = 21)	class II cavities of amalgam (n = 21)	class I cavities of composite resin (n = 13)	class II cavities of composite resin (n = 13)
Total aerobic bacteria	5.43 ± 0.35	5.45 ± 0.51	5.35 ± 0.76	5.41 ± 0.52
Total streptococci	5.25 ± 0.72	5.15 ± 0.45	5.16 ± 0.65	5.24 ± 0.98
*S. mutans*	4.99 ± 0.56	4.94 ± 0.89	4.85 ± 1.08	4.95 ± 1.12
Actinomyces	4.25 ± 1.14	4.24 ± 0.84	4.16 ± 0.37	4.27 ± 1.22
Lactobacilli	5.06 ± 0.60	5.12 ± 0.71	4.90 ± 1.17	5.07 ± 0.62
Neisseriae	4.92 ± 0.87	4.93 ± 0.88	4.74 ± 0.46	4.85 ± 0.92
				
Total anaerobic bacteria	5.91 ± 0.47	5.68 ± 0.79	5.58 ± 0.94	5.64 ± 0.81
Prevotella	5.31 ± 1.55	5.25 ± 1.72	5.33 ± 1.11	5.29 ± 0.72
No-melaninogenicus				
*Prevotella melaninogenicus*	5.21 ± 2.15	5.19 ± 1.48	5.11 ± 1.63	5.17 ± 2.24
Porphyromonas	4.25 ± 1.06	4.22 ± 1.00	4.15 ± 1.89	4.21 ± 0.98
Veillonella	5.33 ± 0.64	5.34 ± 0.82	5.29 ± 1.28	5.32 ± 1.15
Fusobacterium	4.02 ± 0.59	4.12 ± 0.55	4.32 ± 0.88	4.17 ± 1.01
Peptostreptococcus	4.41 ± 0.47	4.59 ± 0.95	4.50 ± 0.75	4.35 ± 1.76
Capnocytophagas	0.44 ± 1.46	0.41 ± 1.92	0.33 ± 1.76	0.37 ± 1.53

Bacteria colony-forming units were presented as log colony-forming units per ml. The composition of the microbe involved in secondary caries was analyzed using SPSS11.5 software. Log colony-forming units per ml were compared by two-way ANOVA analysis using the Bonferroni method. For each type of bacterium, no significant difference was found among four groups (P > 0.05), but the mean of Log colony-forming units for the detected bacteria showed statistical difference among the detected bacteria within each group (P < 0.05).

**Table 3:** The comparison of detectable percentage of the microbes in the four groups.

Bacteria	class I cavities of amalgam (n = 21)	class II cavities of amalgam (n = 21)	class I cavities of composite resin (n = 13)	class II cavities of composite resin (n = 13)
Streptococci	90.00%	86.25%	89.75%	88.89%
*S. mutans*	85.71	80.95	84.62	84.62
Actinomyces	41.43	38.10	43.85	49.23
Lactobacilli	68.57	67.14	68.46	60.77
Neisseriae	61.75	65.45	68.56	62.36
No-black Prevotella	80.95	71.43	73.85	90.31
Black Prevotella	56.58	51.44	50.49	53.37
Porphyromonas	20.15	22.69	19.38	24.57
Veillonella	95.24	85.71	81.54	92.31
Fusobacterium	27.62	28.57	25.38	28.46
Peptostreptococcus	38.10	28.58	38.46	30.77
Capnocytophaga	15.23	10.11	13.45	17.26

The detection rate of each bacterium in each group was calculated as the percentage ratio of the number of sample which we can idenfied one kind of the bacteria from devided by the number of sample in each group. The detection rate of the bacteria involved in secondary caries was analyzed using SPSS11.5 software. For the differences of detection rate of the bacteria among the four groups, Chi-Square Test was used for analysis. There were no significant differences among the four groups for the detection rate of the detected bacterium (P > 0.05), but within each group the detection rate of each bacterium among the detected bacteria had statistical difference (P < 0.05). *S. mutans*, No-black Prevotella, and Veillonella were isolated from secondary caries biofilm in relatively higher percentages than Fusobacterium and Capnocytophaga (P < 0.05). There were no significant differences among the detection rate of others microbial species (P > 0.05).

## Discussion

### The dominant microflora and bacterial spectrum of secondary caries

In this study, the microflora of secondary caries biofilm around Class I and Class II composite and amalgam fillings mainly included *Prevotella, Veillonella*, *Lactobacilli, Streptococci mutans*, and *Neisseriae*. The next most prevalent group of bacteria included *Actinomyces*, *Peptostreptococcus*, *Fusobacterium*, *and Porphyromonas gingivalis *and occasionally *Capnocytophaga*. The proportion of obligately anaerobic species was much greater than that of facultatively anaerobic species. This bacterial spectrum was similar to the microflora in subgingival plaque of periodontal disease and in the infected root canals with potentially pulp pathogenic microbes [30,31]. Christian Splieth et al., who did similar studies, found that the ratios of aerobic to anaerobic flora were comparable for composite and amalgam fillings with 11.4%/88.6% and 15.4%/84.5%, respectively [26]. Very few studies on anaerobic bacteria in secondary caries have been reported. Gonzalez-Cabezas reported the detection of mutans streptococci in secondary carious lesions using confocal laser scanning microscopy and immunofluorescent techniques [32], with this bacteria detected in 88.9% of the samples analyzed with the immunofluorescent technique. Gonzalea-cabezas reported the distribution of three cariogenic bacteria, mutans streptococci, *Actinomyces naeslundii*, and lactobacilli in secondary carious lesions around amalgam restorations [33]. Fitzgerald RJ reported that there was considerable variation in the numbers and types of microorganisms found in dentine samples from lesions of comparable severity [34]. Mutans streptococci were found in 40% of sites with any degree of caries and in only 3 of the 9 sites with the most caries. The prevalence and number of lactobacilli increased with the degree of caries, they occurred in less than half of the affected dentine samples. Actinomyces occurred in 15 of 32 affected sites but only in 2 of the 9 most active sites with their numbers not exceeding 3 × 10^5 ^CFU/mg. However, all these studies have not reported on the anaerobic bacteria of secondary caries.

The samples used in this study were collected from freshly extracted teeth. The teeth were mostly collected from the patients who had periodontal diseases. The Microflora in saliva of the patients could be responsible for the dominant anaerobic bacteria of periodontal diseases. The oral anaerobic bacteria could get into anaerobic environments such as lacuna or along the tooth-restoration margin. Thus, the high proportion of anaerobic species found in this study may not be bacteria directly involved in secondary caries, but may be due to a different etiological model from primary caries.

In primary caries, microorganisms attach to surfaces and develop biofilms. The process is initiated by demineralization of the tooth surface by organic acids. These acids are the result of fermentation of dietary carbohydrates by the plaque bacteria. As tooth mineral is lost, the proteinase of plaque bacteria causes the secondary destruction of tooth protein. In secondary caries, the bacteria may come from oral environment, which gets into anaerobic environment of lacuna or leakage along the tooth-restoration interface. Destruction of tooth protein may be the first step and contributes to cavity formation. After the formation of cavity, bacterial fermentation of dietary carbohydrates can accumulate and produce more acids.

### The effect of filling material and cavity classification on the flora of secondary caries

According the study of Christian Splieth, the variety of microbes under composite fillings was much greater compared to amalgam [26]. In the composite fillings, there were 34 strains of strictly anaerobic non-spore-forming gram-negative rods, 17 strains of anaerobic or facultative anaerobic non-spore-forming gram-positive rods, 9 strains of anaerobic gram-positive cocci, and 2 strains of anaerobic gram-negative cocci. In the amalgam, there was 1 strain of strictly anaerobic non-spore-forming gram-negative rods, 7 strains of anaerobic or facultative anaerobic non-spore forming gram-positive rods, and 3 strains of anaerobic gram-positive cocci. More species and higher quantities of lactobacilli were isolated from composite fillings. Differences between bacterial colonization under composite and amalgam were statistically significant for anaerobic rods (p < 0.05) but not for aerobic rods, anaerobic, and aerobic cocci due to their lower numbers. However, in this study we found no significant differences among the four groups according to the geometric mean of the detected bacteria (P > 0.05).

Other studies reported that bacterial colonization under amalgam of secondary caries was similar to that of flora of carious dentin or carious plaque [35,36] with anaerobic and facultative anaerobic gram-positive rods dominating. This distribution was also present in secondary carious lesions around amalgam restorations [32]. Mejare et al. detected a bacterial spectrum under composite fillings similar to dental plaques with *Actinomyces spp*. and streptococci dominating [37,38]. This bacterial composition could be due to the short persistence of the restorations in the oral cavity in their study. Many studies have focused on antibacterial activity of restorative dental biomaterials [39]. Long-term clinical trials are necessary to determine whether the antimicrobial effects of dental materials are able to reduce the risk of secondary caries formation [40].

In the present study, for each type of bacterium, no significant difference could be found among four groups based upon the geometric mean of the detected bacteria (P > 0.05), the percent of the detected bacteria (P > 0.05), and the prevalence of the detected bacteria (P > 0.05). The results may suggest that the microleakage or cracking is one of the factors for the ecological niche of microorganisms. The anaerobic environment of deep layers of lesions as a result of microleakage along the tooth-restoration interface favors the growth of anaerobes. Thus, the microflora of recurrent caries may be developed as a result of microleakage along the tooth-restoration interface.

## Conclusions

This article is an informative analysis of the microflora around dental restorations. The proportion of obligatory anaerobic species was much greater than facultative anaerobic species in the biofilm of secondary caries. Statistically, the kinds of restoration materials and location of caries have no significant effects on the composition of the microflora.

## Competing interests

In the past five years I have not received any reimbursements, fees, funding, or salary from an organization that may in any way gain or lose financially from the publication of this manuscript, either now or in the future.

I have not held any stocks or shares in an organization that may in any way gain or lose financially from the publication of this manuscript, either now or in the future.

I have not held nor are currently applying for any patents relating to the content of the manuscript. I have not received any reimbursements, fees, funding, or salary from an organization that holds or has applied for patents relating to the content of the manuscript.

I have no other financial competing interests.

There are no other non-financial competing interests (political, personal, religious, ideological, academic, intellectual, commercial or any other) to declare in relation to this manuscript.

## Authors' contributions

SM was responsible for the majority of the study design, bench work and data analysis. WB participated in the clinical sample collection and statistical analysis, and also contributed to the interpretation of the final results. GL and WB performed statistical analysis. JW collected most of the clinical samples. ML participated in the study design, data interpretation and helped draft the manuscript. All authors have read and approved the final manuscript.

## Pre-publication history

The pre-publication history for this paper can be accessed here:

http://www.biomedcentral.com/1471-2334/10/241/prepub
